# Relationship between individual and country-level socio-economic background, USMLE step scores, and demographics of international medical graduates and residency match results

**DOI:** 10.1186/s12909-024-05052-7

**Published:** 2024-02-01

**Authors:** Daria D. Hunter, Ronna L. Campbell, Aidan F. Mullan, Joel R. Anderson, James L. Homme

**Affiliations:** 1 Department of Emergency Medicine, Mayo Clinic, Generose G-410, 200 2nd Stree, t, Rochester, MN 55905 USA; 2 Department of Quantitative Health Sciences, Division of Clinical Trials and Biostatistics, Mayo Clinic, Rochester, MN USA

**Keywords:** Medical education, Residency match, International medical graduates

## Abstract

**Purpose:**

Twenty five percent of practicing physicians in the US are International Medical Graduates (IMGs) – physicians who completed their medical school training outside of the United States and Canada. There are multiple studies demonstrating higher socio-economic background is associated with medical school matriculation in the US. However, despite a substantial prevalence of IMGs in the American healthcare system, studies of the association between demographics, socio-economic background, and securing a residency position in the match are lacking.

**Methods:**

We created a survey with questions on residency match-related data and information on personal socio-economic background. An invitation to participate in the study was sent to all IMGs that applied to the included residency programs after the conclusion of the 2022 residency match. We used multivariable logistic regression to compare survey responses to the odds of securing a residency match.

**Results:**

The total number of survey respondents was 744 (response rate 15.1%). We found that younger age, higher United States Medical License Examination (USMLE) scores, higher-income country of origin (including the United States), fewer match attempts, applying to fewer specialties, having parents with college degree or higher, and coming from higher-than-average or lower-than-average family income were independently associated with increased odds of matching. Gender, personal income, and visa status did not demonstrate significant associations with residency match.

**Conclusions:**

Residency match is a significant expense for IMGs, especially for those from lower-income countries. International applicants from higher socio-economic backgrounds might have advantages in securing medical residency positions in the United States when controlling for other variables.

**Supplementary Information:**

The online version contains supplementary material available at 10.1186/s12909-024-05052-7.

## Introduction

Twenty five percent of practicing physicians in the US are International Medical Graduates (IMGs) – physicians who completed their medical school training outside of the United States and Canada [1]. The percentage of first year residency positions occupied by IMGs varies from year to year. In the 2022 main residency match, 19.5% of all offered positions matched IMGs [2]. IMGs are traditionally classified in two separate categories: US IMGs and non-US IMGs. US IMGs are defined as US citizens who graduated international medical school [2]. In general, US IMGs are US citizens who typically complete a 4-year undergraduate degree (usually completed in the US or Canada) and 4 years of medical school (completed outside of the US and Canada by definition). Non-US IMGs are usually foreign nationals who were born and raised abroad. While a few countries have an educational system similar to the US with undergraduate training preceding medical school, the vast majority of non-US IMGs begin medical training directly after high school with entry into 6 years of medical school without obtaining an undergraduate degree. Physicians who completed their medical school training in the US or Canada are not considered IMGs regardless of their citizenship status.

To secure a residency position, all IMGs must go through the process of obtaining Educational Commission for Foreign Medical Graduates (ECFMG) certification and most use the National Residency Matching Program (NRMP). ECFMG is the only agency facilitating United States Medical License Exams (USMLE), confirming medical school diplomas, and English proficiency for IMGs. In order to become ECFMG certified, an IMG must successfully complete USMLE Step 1 exam, USMLE Step 2 Clinical Knowledge (CK) exam, a specially designed English proficiency exam, possess an ECFMG-validated medical school diploma, and satisfy certain clinical experience criteria which is also confirmed by ECFMG (https://www.ecfmg.org/).

The residency match process is the same for IMG and US medical students and physicians. A total of 42,549 active applicants participated in the 2022 Main Residency Match. Of those, approximately 30% were IMGs: almost 20% non-US IMGs and just over 10% US IMGs [2].

There are multiple studies demonstrating that higher socio-economic background is associated with medical school matriculation in the US [3–5]. According to the 2018 AAMC report, the percentage of matriculating US medical students reporting parental income totals that fall in the top two household income quintiles ranged from 76 to 79% between 2007 and 2017. Results also show that only 5% of all matriculants who provided parental income data in the 2017 questionnaire were in the lowest household-income quintile (annual income of $1,000–24,002), whereas 24% were in the top 5% (annual income of > $225,251) [5]. AAMC also provides data on average age of medical school matriculation – 23–24 years, and gender balance – 51% of US medical students are females [6, 7]. However, despite a substantial prevalence of IMGs in the American healthcare system, studies of the association between demographics, socio-economic background, and securing a residency position through the match are lacking. Little information exists on IMG demographics or socio-economic background. There is, however, good data on IMG USMLE Step scores based on 2022 NRMP report. 'Mean USMLE Step 1 and Step 2 CK scores were approximately 10 points higher for matched US and non-US IMGs comparing to their unmatched counterparts. In addition, matched non-US IMGs scored 10 points higher than US IMGs counterparts (Mean USMLE Step 1 scores for matched and unmatched: US IMGs 222 vs 214: non-US IMGs 235 vs 226. Mean USMLE Step 2 CK scores for matched and unmatched: US IMGs 233 vs 225; non-US IMGs 243 vs 234.) [2]. As of January 2022 USMLE Step 1 score is now pass / fail leaving program directors with fewer numerical characteristics to aid interview selection and ranking.

Little data exists on how much IMGs spend in order to match in US-based medical residency. The cost of medical education for US medical students is well-known and studied by organizations such as AAMC [8]. There are, unfortunately, no such studies to evaluate cost of an attempted residency match to IMGs especially in relation to their income. We know from the Federal State Medical Board (FSMB) census that the largest number of licensed IMGs have graduated from schools in India (23%), followed by the Caribbean (18%), Pakistan (6%), the Philippines (6%) and Mexico (5%) (Caribbean region is the largest supplier of US IMGs) [9, 10]. India and Pakistan are low-middle income countries with GDP of $1,900 and $1,193 per capita, respectively. Philippines and Mexico are upper-middle income countries with GDP per capita of $3,298 and $8,346, respectively. We estimate the cost of an attempted residency match process for an IMG is approximately $14,156 (Table 1) not including cost of medical education in their home countries. Thus, for the 4 countries supplying the largest proportions of non-US IMGs, the estimated relative cost of residency match is 20 months of average annual income for Mexico, 52 months for Philippines, 90 months for India, and 142 months for Pakistan [11].

**Table 1:** Approximate match cost for an IMG (cost of medical school is not included)

Category	Items and prices	Estimated Cost
ECFMG certification	ECFMG certification fee = $160^a^ USMLE Step 1 = $985^a^ USMLE Step 2 CK = $985^a^ International test delivery surcharge for Step 1 = $185^a^ International test delivery surcharge for Step 2 CK = $210^a^ English proficiency exam (OET) = $455^b^ Medical school transcript = $250^a^ ECFMG pathway fee = $925^a^	$4,155
USMLE exam preparation materials	UWorld Step 1 90 days access = $399^c^ UWorld Step 2 CK 90 days access = $399^c^ First Aid Step 1 book = $49.50^d^ First Aid Step 2 CK book = $49.50^d^ USMLE mock exam = $60 per exam^e^	$1,257 including 3 USMLE Step 1 and 3 USMLE Step 2 CK mock exams
US clinical experience	Clinical rotation fee = $1,455 per rotation^f^ Flights = $1,250 round trip^g^ Accommodation = $1,000 per month^h^	$6,160 price for 2 months of US clinical experience
ERAS application to 100 programs	ERAS token = $165^a^ USMLE transcript fee = $80^a^ Application fee to 100 programs = $2,339^i^	$2,584
Total		$14,156

^a^ECFMG

^b^Occupational English Proficiency (OET)

^c^UWorld

^d^First Aid

^e^National Board of Medical Examiners (NBME)

^f^Price of “budget-friendly” internal medicine rotation on “AMOppotrunities” – one of the largest commercial clinical rotation providers advertised on American Medical Association website

^g^Based on the cheapest available round flight from New Deli to New York City, NY (LOT airlines) – searched on 10/13/2022

^h^Based on cheapest available room in Chicago, IL on “Airbnb” website for 4 weeks – searched on 10/13/2022

^i^ERAS (if applying to one specialty)

As evident from the above data, the average relative cost of residency match related expenses is substantial for most IMGs, especially for those from lower income countries. Our study objective was to characterize the relationship between IMG demographic, socio-economic, and academic factors, and the residency match success.

## Methods

We created a survey with questions on residency match-related data and information related to personal socio-economic background. The complete survey is available as Supplemental Digital Appendix 1. The study was approved by the Mayo Clinic Educational Research Committee (ERC) and was deemed to be exempt from the Mayo Clinic Institutional Review Board (IRB) review. Participation was completely voluntary, and no incentives were offered for participation. All study subjects expressed informed consent for use of their residency application data, including contact information, for research purposes while registering on the Electronic Residency Application System (ERAS). We contacted potential study subjects by email requesting their participation in the study by completing the survey. Consent was implied as only the respondents that wished to participate in the study completed the survey. We used The Checklist for Reporting Results of Internet E-Surveys (CHERRIES) as a reporting guideline for our study [12]. All methods were carried out in accordance with relevant guidelines and regulations.

The study was conducted in a large academic institution with three main teaching hospitals in Florida, Minnesota, and Arizona as well as 3 smaller teaching facilities within Minnesota and Wisconsin. The institution trains more than 1,700 GME trainees at any given time. We identified residency programs with the greatest number of IMG resident physicians, including anesthesia, family medicine, general surgery, internal medicine, pathology, and pediatrics [2]. We also included the emergency medicine program due to the relatively large percent of IMG residents compared to the national average for emergency medicine residencies. In compliance with ERAS policy, an invitation to participate in the study was sent to all IMGs (US and non-US) that applied to the included residency programs after the conclusion of the 2022 residency match. Individuals who provided consent were included in the study.

Surveys were distributed using the closed RedCap system generating unique links for each individual email preventing duplicate entries. A reminder email was sent 4 weeks after the first invitation. We only collected data that could not be used to identify individual respondents and stored all responses within our secure firewall system. World Bank data on average income level for country of origin was manually assigned to each respondent. There was a very small number of incomplete questionnaires, and they were included in the final analysis.

Survey responses were divided into two groups (US IMG and Non-US IMG) based on reported country of origin. This distinction was chosen since residency programs often use a similar classification system due to differences in training, visa requirements, and cultural and linguistic background.

The primary outcome of interest was a successful match to a residency in the United States (not necessarily to our institution). Secondary outcomes included match to top-3 ranked programs as defined by the NRMP (one of the applicant’s 3 most desirable programs that they placed at the top of their rank order list), and match to preferred specialty. Multivariable logistic regression was used to compare survey responses (age, gender, visa status, USMLE scores, number of USMLE attempts, parental education and occupation, number of previous match rounds, family income and personal income, relative to people living in the same city and country, country-of-origin income level according to World Bank, number of specialties applied, and amount spent for match in months of salary) from the residency application process to the odds of securing a residency match. Differences between US and non-US IMGs were assessed using interaction terms included in the regression model.

## Results

We received a total of 5,773 email addresses of IMGs who applied to the residency programs of interest from the residency programs we sent data request to. After accounting for duplicates, we had 4,917 unique emails. The total number of survey respondents was 744 (15.1%). Among the 744 survey respondents, there were 545 (73.3%) who reported securing a residency match, including 104 (87.4%) of 119 US graduates and 441 (70.6%) of 625 non-US graduates. Regarding USMLE Step scores, median Step 1 score and step 2 score for matched US IMGs were 220–229 and 230–239, respectively, and for match and unmatched non-US IMGs were 230–239 and 240–249, respectively. Forty-eight percent of respondents were female, mean age was 29.7 years, 59.2% required visa sponsorship (Table 2). Eighty-three percent of respondents had at least one parent with a college degree or higher, and 29.6% had at least one parent working in healthcare. Almost half (48.7%) of respondents reported being from “higher-than-average” or “significantly higher-than-average” income families. And 52.9% were from medium–high or high-income countries.

**Table 2:** International medical graduate survey results

	**Overall (*N* = 744)**		**US Graduates (*n* = 119)**		**Non-US Graduates (*n* = 625)**	
	**Unmatched (*n* = 199)**	**Matched (*n* = 545)**	**Unmatched (*n* = 15)**	**Matched (*n* = 104)**	**Unmatched (*n* = 184)**	**Matched (*n* = 441)**
**Demographics**						
**Age**, years						
Mean (SD)	32.1 (6.4)	28.9 (3.9)	34.3 (5.1)	30.2 (3.9)	32.0 (6.4)	28.6 (3.8)
Median (Q1, Q3)	31.0(28.0, 35.0)	28.0 (26.0, 31.0)	34.0 (30.5, 39.0)	30.0 (27.8, 32.0)	30.0 (27.5, 35.0)	28.0 (26.0, 30.0)
**Gender**, n (%)						
Female	102 (52.3%)	257 (47.4%)	7 (46.7%)	41 (39.4%)	95 (52.8%)	216 (49.3%)
Male	93 (47.7%)	282 (52.0%)	8 (53.3%)	63 (60.6%)	85 (47.2%)	219 (50.0%)
Other	0 (0.0%)	3 (0.6%)	0 (0.0%)	0 (0.0%)	0 (0.0%)	3 (0.7%)
**Visa Status**, n (%)						
US Citizen	44 (22.6%)	145 (26.7%)	14 (93.3%)	103 (100.0%)	30 (16.7%)	42 (9.5%)
US Permanent	18 (9.2%)	49 (9.0%)	0 (0.0%)	0 (0.0%)	18 (10.0%)	49 (11.1%)
Resident						
Employment	14 (7.2%)	19 (3.5%)	1 (6.7%)	0 (0.0%)	13 (7.2%)	19 (4.3%)
Authorization						
Document (EAD)						
Holder						
Requiring Visa	114 (58.5%)	327 (60.1%)	0 (0.0%)	0 (0.0%)	114 (63.3%)	327 (74.1%)
Sponsorship						
Other	5 (2.6%)	4 (0.7%)	0 (0.0%)	0 (0.0%)	5 (2.8%)	4 (0.9%)
**Need for Visa Sponsorship**, n (%)						
Not Requiring Visa	85 (42.7%)	218 (40.0%)	15 (100.0%)	104 (100%)	70 (38.0%)	114 (25.9%)
Sponsorship						
Requiring Visa Sponsorship	114 (57.3%)	327 (60.0%)	0 (0.0%)	0 (0.0%)	114 (62.0%)	327 (74.1%)
**Socio-economic background**						
**Any Parent with College Degree (or Higher),** n (%)						
Under College	37 (19.1%)	80 (14.7%)	3 (20.0%)	26 (25.0%)	34 (19.0%)	54 (12.2%)
College or Higher	157 (80.9%)	465 (85.3%)	12 (80.0%)	78 (75.0%)	145 (81.0%)	387 (87.8%)
**Any Parent Working in Healthcare,** n (%)						
Non-Healthcare	150 (77.3%)	374 (68.8%)	11 (73.3%)	75 (72.8%)	139 (77.7%)	299 (67.8%)
Healthcare	44 (22.7%)	170 (31.2%)	4 (26.7%)	28 (27.2%)	40 (22.3%)	142 (32.2%)
**Family Income (Compared to City Growing Up),** n (%)						
Lower	38 (19.7%)	102 (18.8%)	5 (33.3%)	28 (26.9%)	33 (18.5%)	74 (16.8%)
Average	83 (43.0%)	162 (29.8%)	6 (40.0%)	33 (31.7%)	77 (43.3%)	129 (29.3%)
Higher	72 (37.3%)	280 (51.5%)	4 (26.7%)	43 (41.3%)	68 (38.2%)	237 (53.9%)
**Personal Income (Compared to Current City),** n (%)						
Lower	84 (44.0%)	243 (45.2%)	9 (60.0%)	68 (66.0%)	75 (42.6%)	175 (40.2%)
Average	61 (31.9%)	160 (29.7%)	4 (26.7%)	24 (23.3%)	57 (32.4%)	136 (31.3%)
Higher	46 (24.1%)	135 (25.1%)	2 (13.3%)	11 (10.7%)	44 (25.0%)	124 (28.5%)
**Country of Origin Income Level,** n (%)						
Low	12 (6.0%)	25 (4.6%)	0 (0.0%)	0 (0.0%)	12 (6.5%)	25 (5.7%)
Lower middle	101 (50.8%)	192 (35.2%)	0 (0.0%)	0 (0.0%)	101 (54.9%)	192 (43.5%)
Upper middle	38 (19.1%)	126 (23.1%)	0 (0.0%)	0 (0.0%)	38 (20.7%)	126 (28.6%)
High	39 (19.6%)	191 (35.0%)	15 (100.0%)	104 (100.0%)	24 (13.0%)	87 (19.7%)
Unclassified	9 (4.5%)	11 (2.0%)	0 (0.0%)	0 (0.0%)	9 (4.9%)	11 (2.5%)
**Investment in Match Process,** n (%)						
More than 1 year of income	110 (57.0%)	193 (35.9%)	6 (40.0%)	21 (20.8%)	104 (58.4%)	172 (39.4%)
Less than 1 year of income	35 (18.1%)	119 (22.1%)	5 (33.3%)	16 (15.8%)	30 (16.9%)	103 (23.6%)
Less than 6 months of income	40 (20.7%)	189 (35.1%)	3 (20.0%)	48 (47.5%)	37 (20.8%)	141 (32.3%)
Less than 1 month of income	8 (4.1%)	37 (6.9%)	1 (6.7%)	16 (15.8%)	7 (3.9%)	21 (4.8%)
**Academics and match-related data**						
**USMLE Step 1 Score**, n (%)						
194–209	48 (25.4%)	49 (9.0%)	8 (53.3%)	15 (14.4%)	40 (23.0%)	34 (7.7%)
210–219	44 (23.3%)	64 (11.8%)	3 (20.0%)	19 (18.3%)	41 (23.6%)	45 (10.3%)
220–229	33 (17.5%)	83 (15.3%)	2 (13.3%)	21 (20.2%)	31 (17.8%)	62 (14.1%)
230–239	32 (16.9%)	111 (20.4%)	2 (13.3%)	24 (23.1%)	30 (17.2%)	87 (19.8%)
240–249	12 (6.3%)	115 (21.2%)	0 (0.0%)	17 (16.3%)	12 (6.9%)	98 (22.3%)
250–260	17 (9.0%)	95 (17.5%)	0 (0.0%)	7 (6.7%)	17 (9.8%)	88 (20.0%)
> 260	3 (1.6%)	26 (4.8%)	0 (0.0%)	1 (1.0%)	3 (1.7%)	25 (5.7%)
**USMLE Step 2 CK Score**, n (%)						
< 209	6 (3.1%)	1 (0.2%)	2 (13.3%)	0 (0.0%)	4 (2.3%)	1 (0.2%)
209–219	52 (27.2%)	26 (4.8%)	3 (20.0%)	12 (11.5%)	49 (27.8%)	14 (3.2%)
220–229	42 (22.0%)	74 (13.6%)	3 (20.0%)	20 (19.2%)	39 (22.2%)	54 (12.3%)
230–239	27 (14.1%)	105 (19.3%)	4 (26.7%)	25 (24.0%)	23 (13.1%)	80 (18.2%)
240–249	29 (15.2%)	129 (23.7%)	1 (6.7%)	27 (26.0%)	28 (15.9%)	102 (23.2%)
250–259	23 (12.0%)	119 (21.9%)	2 (13.3%)	16 (15.4%)	21 (11.9%)	103 (23.4%)
> 260	12 (6.3%)	90 (16.5%)	0 (0.0%)	4 (3.8%)	12 (6.8%)	86 (19.5%)
**Recommendation Letters from US Physicians**, n (%)						
0	26 (13.5%)	29 (5.3%)	0 (0.0%)	0 (0.0%)	26 (14.7%)	29 (6.6%)
1	15 (7.8%)	42 (7.7%)	0 (0.0%)	2 (1.9%)	15 (8.5%)	40 (9.1%)
2	19 (9.9%)	72 (13.2%)	1 (6.7%)	8 (7.7%)	18 (10.2%)	64 (14.5%)
3	49 (25.5%)	149 (27.3%)	4 (26.7%)	29 (27.9%)	45 (25.4%)	120 (27.2%)
More Than 3	83 (43.2%)	253 (46.4%)	10 (66.7%)	65 (62.5%)	73 (41.2%)	188 (42.6%)
**Previous Match Rounds**, n (%)						
0	66 (34.0%)	372 (68.4%)	7 (46.7%)	67 (65.0%)	59 (33.0%)	305 (69.2%)
1	78 (40.2%)	139 (25.6%)	4 (26.7%)	30 (29.1%)	74 (41.3%)	109 (24.7%)
2	26 (13.4%)	21 (3.9%)	1 (6.7%)	5 (4.9%)	25 (14.0%)	16 (3.6%)
3	6 (3.1%)	5 (0.9%)	0 (0.0%)	1 (1.0%)	6 (3.4%)	4 (0.9%)
More Than 3	18 (9.3%)	7 (1.3%)	3 (20.0%)	0 (0.0%)	15 (8.4%)	7 (1.6%)
**Number of Specialties Applied,** n (%)						
1	101 (53.4%)	381 (71.3%)	6 (40.0%)	62 (60.2%)	95 (54.6%)	319 (74.0%)
2	61 (32.3%)	122 (22.8%)	6 (40.0%)	31 (30.1%)	55 (31.6%)	91 (21.1%)
3	24 (12.7%)	27 (5.1%)	2 (13.3%)	9 (8.7%)	22 (12.6%)	18 (4.2%)
4	2 (1.1%)	2 (0.4%)	0 (0.0%)	1 (1.0%)	2 (1.1%)	1 (0.2%)
5	1 (0.5%)	1 (0.2%)	1 (6.7%)	0 (0.0%)	0 (0.0%)	1 (0.2%)
More Than 5	0 (0.0%)	1 (0.2%)	0 (0.0%)	0 (0.0%)	0 (0.0%)	1 (0.2%)
**Total Interview Invitations**						
Mean (SD)	2.7 (4.1)	10.6 (8.2)	3.2 (3.1)	14.1 (11.6)	2.6 (4.1)	9.8 (7.0)
Median (Q1, Q3)	1.0 (0.0, 3.8)	9.0 (5.0, 14.0)	2.0 (1.0, 5.5)	12.0 (6.0, 20.0)	1.0 (0.0, 3.0)	8.0 (5.0, 14.0)
**Invitations in Preferred Specialty**						
Mean (SD)	2.2 (3.5)	9.6 (9.2)	1.9 (2.1)	12.4 (15.0)	2.2 (3.6)	8.9 (7.0)
Median (Q1, Q3)	1.0 (0.0, 3.0)	7.0 (4.0, 13.0)	1.0 (0.0, 3.0)	7.0 (3.0, 17.5)	1.0 (0.0, 3.0)	7.0 (4.0, 12.0)
**Interviews Attended**						
Mean (SD)	2.6 (3.9)	9.9 (6.9)	3.3 (3.2)	12.0 (8.6)	2.6 (4.0)	9.4 (6.3)
Median (Q1, Q3)	1.0 (0.0, 3.2)	9.0 (5.0, 14.0)	2.0 (1.0, 5.8)	11.0 (6.0, 17.0)	1.0 (0.0, 3.0)	8.0 (5.0, 13.0)
**Programs Ranked**						
Mean (SD)	3.3 (6.4)	9.1 (6.3)	6.2 (12.5)	10.8 (7.6)	3.0 (5.6)	8.7 (5.9)
Median (Q1, Q3)	1.0 (0.0, 4.0)	8.0 (4.0, 13.0)	2.0 (1.0, 5.5)	10.0 (5.0, 15.0)	1.0 (0.0, 4.0)	8.0 (4.0, 12.0)

Among all respondents a little over half were from high and middle-high income countries. Among non-US IMGs only 17.6% were from high and 26.2% from middle-high income countries. Most non-US IMGs (46.8%) were from lower-middle, and only 5.7% from low-income countries.

In a multivariable model comparing match outcomes of IMGs for the full cohort, including both US and non-US IMGs (Table 3), we found that older age was associated with a 9% (OR = 0.91, 95% CI: 0.86–0.95, *p* < 0.001) per year decreased odds of matching to a residency program, an 8% (OR = 0.92, 95% CI: 0.88–0.96, *p* < 0.001) per year decreased odds of matching into an applicant’s three highest ranked programs (top-3 ranked), and a 7% (OR = 0.93, 95% CI: 0.89–0.98, *p* = 0.003) per year decreased odds of matching to a preferred specialty. Applicants from lower-than-average and higher-than-average income families were both more likely to match when compared to applicants from average-income families with 114% (OR = 2.14, 95% CI: 1.20–3.87, *p* = 0.011) and 65% (OR = 1.65, 95% CI: 1.02–2.66, *p* = 0.041) increase in odds of matching, respectively. Graduates from countries with high or upper middle income were 66% more likely to match (OR = 1.66, 95% CI: 1.06–2.61, *p* = 0.027). Applicants who identified their country of origin as United States were 161% more likely to match (OR = 2.61, 95% CI: 1.22–5.81, *p* = 0.016) compared to IMGs from other countries.

**Table 3:** Multivariable predictors for success in residency match, full cohort (*N* = 744)

**Predictor**	**Predictors of residency match**			**Predictors of match to preferred specialty**			**Predictors of match to top-3 chosen programs**		
	**Odds Ratio**	**95% CI**	***P*-Value**	**Odds Ratio**	**95% CI**	***P*-Value**	**Odds Ratio**	**95% CI**	***P*-Value**
**Demographics**									
**Age**									
Per Year	0.91	(0.86, 0.95)	< 0.001	0.93	(0.89, 0.98)	0.003	0.92	(0.88, 0.96)	< 0.001
**Gender**									
Not Male	Reference			Reference			Reference		
Male	0.98	(0.65, 1.49)	0.93	0.77	(0.52, 1.14)	0.31	0.84	(0.60, 1.18)	0.31
**Visa Status**									
Not Requiring Sponsorship	Reference			Reference			Reference		
Requiring Sponsorship	0.81	(0.49, 1.34)	0.42	1.16	(0.72, 1.86)	0.54	0.80	(0.52, 1.22)	0.30
**Socio-economic background**									
**Parent Education**									
No College Degree	Reference			Reference			Reference		
College Degree or Higher	1.40	(0.79, 2.44)	0.25	1.08	(0.64, 1.82)	0.77	1.36	(0.85, 2.18)	0.20
**Parent Occupation**									
Not Healthcare	Reference			Reference			Reference		
Healthcare	1.29	(0.81, 2.08)	0.30	1.00	(0.66, 1.54)	0.99	1.05	(0.72, 1.52)	0.81
**Family Income**									
Average	Reference			Reference			Reference		
Lower	2.14	(1.20, 3.87)	0.011	1.71	(1.00, 2.95)	0.052	1.35	(0.83, 2.19)	0.22
Higher	1.65	(1.02, 2.66)	0.041	1.44	(0.93, 2.24)	0.10	1.22	(0.83, 1.81)	0.31
**Personal Income**									
Average	Reference			Reference			Reference		
Lower	1.15	(0.71, 1.86)	0.58	0.92	(0.59, 1.44)	0.73	1.05	(0.71, 1.55)	0.82
Higher	1.53	(0.86, 2.76)	0.15	1.46	(0.85, 2.53)	0.17	1.42	(0.89, 2.27)	0.15
**Country of origin**									
Not United states	Reference			Reference			Reference		
United States	2.61	(1.22, 5.81)	0.016	1.53	(0.80, 2.96)	0.20	1.18	(0.66, 2.12)	0.58
**Country of Origin Income**									
Low or Lower Middle	Reference			Reference			Reference		
High or Upper Middle	1.66	(1.06, 2.61)	0.027	1.42	(0.93, 2.16)	0.11	1.29	(0.89, 1.87)	0.18
**Investment in Match**									
Under 1 Month of Income	Reference			Reference			Reference		
1–6 Months of Income	1.71	(0.58, 4.57)	0.30	1.08	(0.41, 2.65)	0.87	1.53	(0.72, 3.23)	0.26
6–12 Months of Income	1.22	(0.41, 3.33)	0.70	0.67	(0.25, 1.69)	0.41	1.34	(0.61, 2.92)	0.46
More than 1 Year of Income	0.67	(0.23, 1.72)	0.42	0.57	(0.22, 1.37)	0.22	1.03	(0.48, 2.16)	0.94
**Academics and match-related data**									
**USMLE Step 1 Score**									
Per 10-point Increase	1.23	(1.04, 1.46)	0.017	1.18	(1.01, 1.38)	0.034	1.02	(0.89, 1.18)	0.73
**USMLE Step 2 CK Score**									
Per 10-Point Increase	1.27	(1.05, 1.53)	0.012	1.11	(0.94, 1.32)	0.23	1.13	(0.97, 1.32)	0.12
**Previous Match Rounds**									
Per Additional Round	0.71	(0.55, 0.91)	0.007	0.67	(0.51, 0.86)	0.002	0.79	(0.62, 0.99)	0.043
**Specialties Applied**									
Per Additional Specialty	0.91	(0.67, 1.23)	0.53	0.42	(0.31, 0.56)	< 0.001	0.71	(0.54, 0.93)	0.012

A 10-point increase in Step 1 score was associated with 23% greater odds of matching (OR = 1.23, 95% CI: 1.04–1.46, *p* = 0.017) and 18% greater odds of matching to preferred specialty (OR = 1.18, 95% CI: 1.01–1.38, *p* = 0.034). A 10-point increase in Step 2 CK score was associated with 27% greater odds of matching (OR = 1.27, 95% CI: 1.05–1.53, *p* = 0.012). Conversely, graduates who had attempted to match previously had decreased odds of matching with each additional match attempt decreasing the odds of successfully matching by 29% (OR = 0.71, 95% CI: 0.55–0.91, *p* = 0.007), decreasing the odds of matching into a top-3 ranked program by 21% (OR = 0.79, 95% CI: 0.62–0.99, *p* = 0.043), and decreasing the odds of matching to preferred specialty program by 33% (OR = 0.67, 95% CI: 0.51–0.86, *p* = 0.002). We found that the larger number of specialties applied was also associated with decreased chances to match to their top-3 positions with 29% decrease per each additional specialty applied (OR = 0.71, 95% CI: 0.54–0.93, *p* = 0.012). Applicants who applied to multiple specialties were also less likely to match to preferred specialty with 58% decrease per each additional specialty (OR = 0.42, 95% CI: 0.31–0.56, *p* < 0.001). Gender, personal income, parent occupation, visa status, and other factors were not significantly associated with residency matching.

When assessing non-US graduates alone we found similar associations between age, USMLE scores, family income level, previous match attempts, number of specialties applied and residency match outcomes (Table 4). In addition, applicants with at least one parent with a college degree or higher were 81% more likely to match to a top-3 ranked program (OR = 1.81, 95% CI: 1.06–3.10, *p* = 0.029). Non-US IMGs from countries with high or upper middle income were again 66% more likely to match (OR = 1.66, 95% CI: 1.06–2.60, *p* = 0.027).

**Table 4:** Predictors for residency match success for non-US graduates (*N* = 625)

**Predictor**	**Predictors of residency match**		**Predictors of match to preferred specialty**		**Predictors match to top-3 chosen programs**	
	**Odds Ratio (95% CI)**	***P*-Value**	**Odds Ratio (95% CI)**	***P*-Value**	**Odds Ratio (95% CI)**	***P*-Value**
**Demographics**						
**Age**						
Per Year	0.91 (0.87, 0.96)	0.001 *	0.93 (0.88, 0.98)	0.005 *	0.92 (0.87, 0.96)	< 0.001 *
**Gender**						
Not Male	Reference					
Male	0.93 (0.60, 1.45)	0.75	0.76 (0.50, 1.15)	0.20	0.81 (0.56, 1.17)	0.27
**Visa Status**						
Not Requiring Sponsorship	Reference					
Requiring Sponsorship	0.81 (0.49, 1.34)	0.42	1.15 (0.71, 1.85)	0.57	0.80 (0.51, 1.23)	0.31
**Socio-economic background**						
**Parent Education**						
No College Degree	Reference					
College Degree or Higher	1.45 (0.79, 2.67)	0.23	1.33 (0.74, 2.38)	0.34	1.81 (1.06, 3.10)	0.029 *
**Parent Occupation**						
Not Healthcare	Reference					
Healthcare	1.32 (0.80, 2.17)	0.27	1.11 (0.70, 1.76)	0.66	1.29 (0.86, 1.94)	0.22
**Family Income**						
Average	Reference					
Lower	2.33 (1.23, 4.37)	0.009 *	2.12 (1.15, 3.90)	0.016 *	1.42 (0.83, 2.44)	0.20
Higher	1.69 (1.03, 2.79)	0.040 *	1.59 (0.99, 2.54)	0.054	1.35 (0.89, 2.05)	0.16
**Personal Income**						
Average	Reference					
Lower	1.12 (0.67, 1.86)	0.68	0.83 (0.51, 1.34)	0.44	0.96 (0.63, 1.48)	0.86
Higher	1.53 (0.84, 2.78)	0.17	1.43 (0.81, 2.52)	0.22	1.31 (0.81, 2.14)	0.27
**Country of Origin Income**						
Low or Lower Middle	Reference					
High or Upper Middle	1.66 (1.06, 2.60)	0.027 *	1.35 (0.89, 2.08)	0.24	1.25 (0.86, 1.83)	0.24
**Investment in Match**						
Under 1 Month of Income	Reference					
1–6 Months of Income	2.03 (0.65, 6.37)	0.22	2.05 (0.72, 5.84)	0.18	2.20 (0.88, 5.47)	0.090
6–12 Months of Income	1.74 (0.55, 5.56)	0.35	1.34 (0.46, 3.87)	0.59	1.84 (0.73, 4.67)	0.20
More than 1 Year of Income	0.85 (0.28, 2.57)	0.12	1.02 (0.37, 2.82)	0.97	1.31 (0.54, 3.18)	0.55
**Academics and match-related data**						
**USMLE Step 1 Score**						
Per 10-point Increase	1.20 (1.01, 1.43)	0.037 *	1.15 (0.98, 1.35)	0.097	1.01 (0.87, 1.16)	0.93
**USMLE Step 2 CK Score**						
Per 10-Point Increase	1.29 (1.06, 1.56)	0.010 *	1.13 (0.95, 1.36)	0.17	1.14 (0.97, 1.35)	0.10
**Previous Match Rounds**						
Per Additional Round	0.71 (0.55, 0.93)	0.014 *	0.66 (0.50, 0.87)	0.004 *	0.77 (0.60, 0.99)	0.041 *
**Specialties Applied**						
Per Additional Specialty	0.90 (0.65, 1.24)	0.51	0.44 (0.32, 0.61)	< 0.001 *	0.70 (0.52, 0.94)	0.018 *

*indicates variables with *P*<0.05 (statistically significant results)

## Discussion

In this study of 744 IMGs applying for the 2022 residency match, we found that younger age, higher USMLE scores, higher-income country of origin (including the United States), fewer match attempts, applying to fewer specialties, having parents with college degree or higher, and coming from higher-than-average or lower-than-average family income were associated with increased odds of matching. Gender, personal income, and visa status did not demonstrate significant associations with residency match.

According to the NRMP in the 2022 residency match 5,048 US IMGs and 7,864 non-US IMGs submitted rank lists [2]. Thus, surveys for this study were sent to 38% of all IMGs who applied to the 2022 match with 5% of all IMGs participating in 2022 residency match responding. To our knowledge, only a limited number of studies focusing on IMGs’ residency match exist. Most of the data comes from the annual NRMP report.. This information is limited to data such as USMLE scores, research publications, work and volunteer experiences, and number of specialties applied. There are, however, papers focusing on specific economic and cultural challenges of IMGs demonstrating that IMGs from more developed countries match to more competitive specialties and residency programs [13, 14].

Our study cohort had a higher proportion of successfully matched applicants (87.3% of US IMGs, 70.5% of non-US IMGs) compared to the total population of applicants per the NRMP which reported a match rate of 61.4% for US IMGs and 58.1% for non-US IMGs in the 2022 residency match. Similarly, our rate of match to the preferred specialty was higher with 68.9% of US IMGs and 63.8% of non-US IMGs matching to their preferred specialty compared to the 2022 match rate to preferred specialty which was 54.8% for US IMGs, 53.5% for non-US IMGs [2]. Despite these differences, we still had a substantial proportion of unmatched non-US IMGs which allowed us to perform a multivariable analysis of factors associated with matching. Other than a higher match rate in our cohort, the rest of the reported variables, including Step scores and percent of female applicants, were similar to national average based on the NRMP data. This suggests that the data likely can be generalized to the other residency programs in the US. Our analysis of US IMGs was limited due to the small number of unmatched applicants in our cohort. Findings demonstrated that graduates from countries with high or upper middle income were more likely to secure residency position, and applicants with at least one parent with a college degree or higher were more likely to match to a program listed in one of the top-3 spots on their rank list.. IMGs from higher income families were also more successful in residency match. This supports our theory that IMG applicants from higher socio-economic backgrounds were more likely to secure residency positions even when accounting for other variables. Higher socio-economic status is also associated with medical school matriculation among American medical students. A 2018 AAMC study of 126,856 1st year US medical students from 1988 through 2017, the top two household income quintiles contributed between 73 and 79% of all US medical school matriculants each year. Interestingly, matriculants in higher-income quintiles were also more likely to be children of parents with at least a bachelor’s degree [5]. Regarding parental education, there are similar findings among US medical students. In 2022 only 21.5% of US medical school matriculants had parents with less than a college degree [15].

Our study did also demonstrate an association between being from a lower-than-average income family with higher chances of matching. While this result seems to contradict another finding of our study, it is possible that both are true. Admittedly it is very difficult to compare between residents of different countries since family income is self-reported and potentially subjective. However, with an increasing focus on diversity, resilience and grit in the residency selection process, applicants from lower-income families may have an advantage in demonstrating these qualities. The true association between family income and residency match success requires further study.

Per our data, age was significantly associated with chances to match, match to preferred specialty, and top-3 programs. Younger applicants do better in all three outcomes. The average age of a first-year medical resident in the US is 29.8 years [16], the average age of matched IMG in our dataset was similar (28.9 years).

In addition to the finding of applicants from higher income countries being 66% more likely to secure residency position, there is another observation. Approximately 10% of the world’s population comes from low-income countries [17]. Among our non-US IMG respondents only 5.7% reported being from a country classified as low-income by the World Bank. This underrepresentation could be due to financial barriers faced by these applicants.

There are programs in the US medical education, such as VSLO (Visiting Student Learning Opportunities) which charge different annual fees depending on a country’s income level [18]. The ECFMG’s fees, however, are the same for every IMG. The fees are lower for IMGs residing in the US because they do not pay an international surcharge for the USMLE exam administration. In addition, USMLE examinations are less likely to be available in low-income countries, requiring applicants from these countries to travel internationally to sit for each exam which further increases the relative cost of the match for them [17]. The COVID-19 pandemic likely widened the gap between IMG applicants from different countries even more, starting with economic damages disproportionally affecting low-income countries and ending with new regulations complicating international travel especially for nationals of countries where Western vaccines are not readily available and those requiring US visas [19, 20].

We found that the increased number of specialties applied to was associated with a decreased odd of matching. This finding is consistent with NRMP reports demonstrating that applicants applying to a higher number of specialties have lower chances of matching. This finding may be due to residency programs perceiving applicants with multi-specialty CVs as having lower commitment to any given specialty. Alternatively, applicants applying to more competitive specialties are more likely to use less competitive specialties as a secondary option. Further specialty-specific studies are needed to analyze these findings.

Visa status was not associated with odds of matching. This is likely because our institution sponsors all types of visas for IMGs, however, this finding may not be generalizable to other institutions where only particular types of visas are sponsored.

To improve access to US graduate medical education for international applicants from lower socio-economic backgrounds, consideration of a sliding scale payment system for the variety of fees associated with the entire process could be introduced. Additional studies are needed to survey larger number of international applicants regarding the financial barriers they experience to entering the US graduate medical education system prior to developing this type of system.

Based on our data, any IMG applying to the NRMP would be advised to put their absolute best application forward the first time rather than “taking a shot” and seeing how they fair. Age was also a significant predictor of match success so waiting many years to apply could offset some of the gains in other areas. For those applicants who are further out from their primary medical training, they may need to find additional ways to connect with or highlight their value to programs to demonstrate how their prior experience is an asset and not a liability, since our data suggests a preference for younger applicants. We acknowledge that this finding could be the result of older applicants having more attempts due to weaker applications within our data set. It is also possible that applicants from lower socio-economic backgrounds are not able to apply shortly after medical school graduation as they might need to work for several years to be able to afford the USMLE and NRMP cost.

### Limitations

This was a retrospective study in order to comply with ERAS policies. Our response rate was relatively low at 15.13% but does represent 5% of all IMGs applying for 2022 residency match. This is still a relatively small sample compared to the number of IMGs applying for residency match each year. An unmeasured confounding is a potential limitation of this study. Due to difficulties with comparing socio-economic characteristics of people from different countries, we had to use subjective variables such as personal perception of the participants of their level of income growing up in comparison to other families in the same city. The match rate of our respondents was higher compared to the total population of applicants per the NRMP which could represent self-selection bias. There is a small chance all associations identified were due to statistical error.

We used contact information provided by the applicants as a part of NRMP. Based on our sample, more than 1/3 of all IMGs applying that year applied to our institution, they also likely applied to hundreds of other programs, and therefore, we do not believe that competitiveness of our institution was a significant limiting factor of this study, although it is a possibility.

## Conclusion

Overall younger applicants, applicants from higher socio-economic backgrounds and applicants with higher USMLE scores were more likely to succeed in residency match process. Costs associated with entering the match are significant for most non-US IMG applicants which could limit participation of individuals from lower income countries. Currently there is no price differential for applicants from different countries based on socioeconomic means. Larger studies are needed to analyze unique challenges faced by international medical graduates in the match process to help inform potential solutions for improved access. Future research is needed to address other aspects of IMG training such as residency experiences, performance, and post-residency employment.

### Supplementary Information

**Additional file 1.** Survey.

## Data Availability

The data that support the findings of this study are available from the ERAS but restrictions apply to the availability of these data, which were used under license for the current study, and so are not publicly available. Data are however available from the corresponding author (Daria Hunter, MD) upon reasonable request and with written permission of the ERAS.
