# Supplementary material for: Relationship between individual and country-level socio-economic background, USMLE step scores, and demographics of international medical graduates and residency match results
Source: BMC Med Educ. 2024 Feb 1;24:108. doi: 10.1186/s12909-024-05052-7 (PMC10835816; doi:10.1186/s12909-024-05052-7)
Supplement: Supplementary file 1 — Additional file 1. Survey. [file 12909_2024_5052_MOESM1_ESM.pdf]

Survey

Question	Answer
How old are you?	Blank field
What is your gender?	Male / female / other
What is your country of origin?	Blank field
In what country did you attend medical school?	Blank field
In what country have you permanently resided over the past 2 years?	Blank field
What will be your visa status by the time of the residency start date?	US citizen / Legal Permanent Resident / EAD holder / requiring visa / other
What kind of US clinical experience have you had?	Official elective / hands-on clinical experience other than elective / hands-off clinical experience (observership) / none / other
How many letters of recommendation from US based physicians do you have?	0 / 1 / 2 / 3 / > 3
How many times have you participated in the match (not including this match season)?	0 / 1 / 2 / 3 / > 3
What is your most recent USMLE Step 1 score?	< 194 / 194-209 / 210-219 / 220-229 / 230-239 / 240-249 / 250-260 / > 260 / none
How many USMLE Step 1 attempts did you have?	1 / 2 / 3 / >3 / Haven't taken Step 1 yet
What is your most recent USMLE Step 2 CK score?	< 209 / 209-219 / 220-229 / 230-239 / 240-249 / 250-260 / > 260 / none
How many USMLE Step 2 CK attempts did you have?	1 / 2 / 3 / >3 / Haven't taken Step 2 CK yet
How would you describe highest level of education completed by your mother (parent 1)?	None / elementary school / high school / some college / college degree / masters / doctorate
What best characterizes your mother's (parent's 1) occupation?	None / Skilled and unskilled labor / Service and sales / Professional or managerial position / Education and research / Law enforcement and army / Government and clerks / Private business
How would you describe highest level of education completed by your father (parent 2)?	None / elementary school / high school / some college / college degree / masters / doctorate

Question	Answer
What best characterizes your father's (parent's 2) occupation?	None / Skilled and unskilled labor / Service and sales / Professional or managerial position / Education and research / Law enforcement and army / Government and clerks / Private business
How would you characterize income level of your parental family in relation to other people living in the same city when you were growing up?	Significantly lower / slightly lower / about the same / slightly higher / significantly higher
How would you characterize your personal income level now in relation to other people living in the same city as you?	Significantly lower / slightly lower / about the same / slightly higher / significantly higher
How much did you invest in preparation to the match process (USMLE exams and study materials, US clinical experience, ERAS applications, etc) not including medical school tuition and fees?	Less than 1 month of your (or your sponsor's) income / less than 6 months of your (or your sponsor's) income / less than 1 year of your (or your sponsor's) income / more than 1 year of your (or your sponsor's) income
How many specialties are you applying to?	Blank field
What is your preferred specialty?	Blank field
How many programs have you applied to?	Blank field
How many total interview invitations have you had?	Blank field
How many interview invitations in your preferred specialty have you had?	Blank field
How many interviews you attended?	Blank field
How many programs you ranked?	Blank field
Did you secure a residency position?	Yes, matched / yes, SOAped / yes, rematched / no / other
Did you get a position in your preferred specialty?	Yes / no / other
What was the number of the program you matched to on your ROL?	Blank field
